# An Audit on the Prevalence of Attention Deficit Hyperactivity Disorder (ADHD) in the General Adult Community Mental Health Team Caseload and Compliance With the National Institute for Health and Care Excellence (NICE) Guidelines on Physical Health monitoring

**DOI:** 10.1192/bjo.2026.11667

**Published:** 2026-06-30

**Authors:** Helen McKaigue, Ryan McNamara

**Affiliations:** 1 NIMDTA, Belfast, United Kingdom; 2 Southern Trust, Armagh, United Kingdom

## Abstract

**Aims:**

- To collect a database of all patients with ADHD in the adult mental health community team.
- Investigate current compliance with following NICE guidelines on physical health monitoring for patients with ADHD taking medication.
- Ensure all patients on medications were under the care of the Physical Health Monitoring Team (PHMT) who undertake the monitoring.
- Make recommendations for improvement in care where guidelines are not being adhered to.

**Methods:**

A database was compiled with patients suffering from ADHD and those on medications were noted.

The following criteria from the NICE guidelines on ADHD management were included:

- Baseline observations (heart rate (HR), blood pressure (BP), height, weight) – we reviewed this for those patients who had commenced ADHD medications less than one year ago.
- 6 monthly weight
- 6 monthly HR and BP
- 6 monthly psychiatry review
- Assessment for side effects at review – This includes cardiovascular issues, emerging tics / abnormal movements, sexual dysfunction, seizures, sleep, worsening behaviour

**Results:**

- 21/31 patients with confirmed ADHD were being medicated for same.
- 10/21 medicated patients were not under the care of the PHMT nor did they have a key worker.
- Medications included forms of methylphenidate or lisdexamfetamine and/or both:9/21 (43%) Concerta XL, 5/21 (24%) Equasym XL, Lisdexamfetamine 6/21 (29%), methylphenidate 3/21 (14%).
- 9/21 (43%) did not receive appropriate baseline observations.
- 14/21 (67%) did not have a 6 monthly weight documented.
- 15/21 (71%) did not have 6 monthly HR/BP checks
- 8/21 (38%) did not have a 6-month psychiatry review.
- Regarding assessment for side-effects, on reviewing clinical letters, 1/21 (5%) of letters did not document any discussion regarding side effects. Variation in side effects discussed was evident with 10/21 (48%) documenting general side effects such as sleep and 8/21 (38%) documenting more specific side effects such as tics or sexual dysfunction.

**Conclusion:**

This audit highlighted that ADHD monitoring was being neglected in a large percentage of patients on our ADHD database and variation in documentation regarding side effects.

- Review led to the following improvements being made:
- We developed an identifiable case load of patients with ADHD which is helpful for future audit and monitoring.
- Patients were referred to the PHMT for follow up of monitoring, helping to improve the likelihood that NICE guidelines will be adhered to.
- A reflection on the need for more specific documentation regarding monitoring of side effects in clinical letters.

